# *In-silico* characterization of *PSEN1* missense variants associated with acne inversa

**DOI:** 10.17912/micropub.biology.002066

**Published:** 2026-07-14

**Authors:** Kate Grissom, Cynthia Stenger, Lydia Uptain

**Affiliations:** 1 Biology, University of North Alabama, Florence, AL, USA; 2 Mathematics, University of North Alabama, Florence, AL, USA

## Abstract

Acne inversa, or hidradenitis suppurativa, is a chronic inflammatory skin disorder that causes painful and reoccurring abscesses. The *PSEN1* gene is responsible for the sequence coding for the presenilin 1 protein, which is part of the gamma-secretase complex and is responsible for cleaving multiple peptides. Acne inversa occurs when the Notch signaling pathway of *PSEN1* is disrupted. Analysis of multiple pathogenicity predictors and simulated aqueous environments of the transmembrane protein in its native state and mutated state through molecular dynamics simulation predicted deleterious effects from swap of tyrosine to cysteine at the highly conserved and buried position 189.

**Figure 1. Characterization of Y189C:** **A)** 3D model of *PSEN1* with transmembrane code generated in YASARA. The positions of Y159C (known pathogenic) and variant of uncertain significance (VUS) Y189C are shown directly outside of the transmembrane region. **B)** *PSEN1* YASARA model. The top right image shows the native type in gray, and the bottom right image shows VUS Y189C in blue, found at the end of an alpha helix region. This YASARA model used a PDB file from UniProt (AF-A0A024R6A3-F1) and the swap feature in YASARA (Ahmad et al., 2025). **C)** ConSurf model of PSEN1 with the darker pink regions indicating higher conservation. VUS Y189C (see arrow) is found to be at level 8 on the conservation scale (Yariv et al., 2023; Landau et al., 2005). **D)** Analysis of in-silico pathogenicity predictor tools SIFT, PolyPhen-2, REVEL, Meta LR, and Mutation Assessor from the Ensembl database (Harrison et al., 2024). Normalized scores reflect predictions of pathogenicity between a known benign variant in green, known pathogenic variant in red, and VUS in blue. The VUS Y189C has an equal or higher pathogenicity score compared to the known pathogenic variant across all predictor tools. **E)** The Root Mean Square Deviation (RMSD) results of the 20 nanosecond (ns) molecular dynamics simulation using a transmembrane model showed the movement of the VUS Y189C (in blue) is significantly closer in deviation to the movement of the pathogenic variant Y159C (in red) when compared to the movement of the the native type (in gray) (Krieger & Vriend, 2015; Krieger & Vriend, 2014; Krieger et al., 2009).

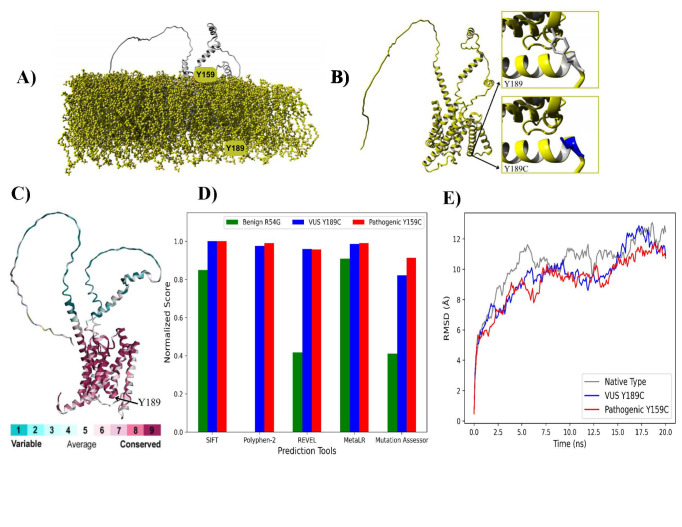



## Description

The *PSEN1* gene contains the sequence coding for the presenilin 1 protein, which is part of the gamma-secretase complex and is responsible for cutting apart other proteins to form peptides (Melnik & Pelwig, 2013a) **.** The gamma-secretase complex is an important part of cell signaling pathways, including processing the amyloid precursor protein (associated with Alzheimer’s disease) and the Notch signaling pathway. Notch signaling pathway disruption due to mutations in *PSEN1* contributes to the expression of acne inversa. The Notch signaling pathway is crucial for the maintenance of the inner and outer root sheath in hair follicles and skin; disruption can result in abnormal skin cells and hair follicle growth, leading to physical abnormalities on the skin (Melnik & Pelwig, 2013b). Acne inversa is recognized as contributing to the lowest quality of life compared to other dermatological disorders such as Dowling-Degos disease, Adams-Oliver syndrome, psoriasis, and atopic dermatitis (Brandão et al., 2021). Diagnosis is associated with an increased comorbidity rate with mental health disorders (e.g. anxiety, depression), a negative body image, and increased emotional difficulty for those diagnosed (Machado et al., 2019; Vellaichamy et al., 2021).

Using data collected from gnomAD, variant Y189C was selected because of its association with acne inversa and location outside the transmembrane ( Figure 1A ) (Bagaria et al., 2022; Chen et al., 2024). The swap from tyrosine to cysteine is a polar to nonpolar swap ( Figure 1B ) that occurs in a highly conserved area ( Figure 1C ). ConSurf conservation analysis confirmed that a mutation in position 189 would be impactful on the structure and function of the protein, as it showed position 189 to be highly conserved and buried ( Figure 1C ) (Yariv et al., 2023; Landau et al., 2005). Pathogenicity predictions from ConSurf have been shown to correlate well with *in vitro* results (Gay et al., 2024). Normalized scores from multiple *in-silico* pathogenicity prediction tools found the variant of uncertain significance (VUS) comparable to a known pathogenic (disease-causing) variant, and dissimilar to a known benign (not known to be disease-causing) variant ( Figure 1D ). A molecular dynamics simulation (MDS) was used to simulate the movement of the native type and VUS, along with a known pathogenic variant of the same amino acid swap in an aqueous environment over 20 ns. Analysis of the MDS results showed similarity in movement of the VUS and known pathogenic variant. A one-tailed paired difference t-test found the average difference in movement between the variant and the known pathogenic significantly less than the difference in movement between the variant and the native (p < 0.001). This indicates that there may be structural impacts similar to the known pathogenic variant that may lead to significant functional changes ( Figure 1E ). These results should motivate future investigations to further characterize unknown variants, leading to more knowledgeable patient diagnosis and the development of treatments or therapies for those with acne inversa.

## Methods

Acne inversa is commonly associated with genetic variations of *PSEN1* ; *PSEN1* was chosen due to the high frequency of related clinical submissions in Simple ClinVar (Perez-Palma et al., 2019). Variants of *PSEN1* were analyzed and the VUS resulting from a missense swap of tyrosine to cysteine at position 189 (Y189C) was chosen for study due to its structural nature, its structural similarities to Y159C, already classified as pathogenic (Bagaria et al., 2022), and its known association with acne inversa [accession ID - rcv001325814.7] (Chen et al., 2024). ConSurf modeling predicted conservation scores for position 189 over 150 homologues (Yariv et al., 2023; Landau et al., 2005). Multiple pathogenicity predictor scores from a known benign, a known pathogenic, and VUS Y189C were normalized to a scale ranging from 0 to 1, 0 indicating more benign and 1 indicating more pathogenic (Harrison et al., 2024). To further investigate the structural impact of the variant, compared to the native type and to a known pathogenic missense swap, molecular dynamics simulations (MDS) were generated. Program inputs for the three-dimensional *PSEN1* structure were formed from the AlphaFold model AF-A0A024R6A3-F1 using the YASARA program (Krieger & Vriend, 2015; Krieger & Vriend, 2014; Krieger et al., 2009). The three-dimensional structures for the unclassified variant Y189C and the pathogenic variant Y159C were formed by replacing the respective residue in the native protein structure (P49768-1) found in UniProt (Ahmad et al., 2025). Molecular dynamics with added transmembrane code inputs were set to simulate the movement of the protein over 20 nanoseconds in an aqueous environment. The RMSD values over time help to visualize structural changes ( Figure 1E ). These methods support further experimental studies to validate these findings in the context of acne inversa pathology.
